# Describing people with cognitive impairment and their complex treatment needs during routine care in the hospital – cross-sectional results of the intersec-CM study

**DOI:** 10.1186/s12877-021-02298-4

**Published:** 2021-07-12

**Authors:** F. Kracht, M. Boekholt, F. Schumacher-Schönert, A. Nikelski, N. Chikhradze, P. Lücker, H. C. Vollmar, W. Hoffmann, S. H. Kreisel, J. R. Thyrian

**Affiliations:** 1 German Center for Neurodegenerative Diseases (DZNE), site Rostock/ Greifswald, Greifswald, Germany; 2 Evangelisches Klinikum Bethel, Campus Bielefeld-Bethel, Division of Geriatric Psychiatry, Universitätsklinikum OWL der Universität Bielefeld, Bielefeld, Germany; 3 Institute of General Practice and Family Medicine (AM RUB), Faculty of Medicine, Ruhr University Bochum (RUB), Bochum, Germany; 4 Institute for Community Medicine, Section of Epidemiology of Health Care and Community Health, University Medicine Greifswald, Greifswald, Germany

**Keywords:** Dementia, Acute hospital care, Cognitive impairment, Germany, RCT, Intersectoral care, Primary care

## Abstract

**Background:**

Cognitive impairment is an important determinant in health care. In the acute hospital setting cognition has a strong impact on treatment and care. Cognitive impairment can negatively affect diagnostics and treatment success. However, little is known about the individual situation and specific risks of people with cognitive impairments during hospital stays. The aim of the present research is to describe and analyze the treatment needs of people with cognitive impairments in acute hospital care.

**Methods:**

The analyses use baseline data of the ongoing multisite, longitudinal, randomized controlled intervention trial intersec-CM (Supporting elderly people with cognitive impairment during and after hospital stays with Intersectoral Care Management), which recruited 402 participants at baseline. We assessed sociodemographic aspects, cognitive status, functional status, frailty, comorbidities, level of impairment, formal diagnosis of dementia, geriatric diagnoses, delirium, depression, pharmacological treatment, utilization of health care services and health care related needs.

**Results:**

The sample under examination had been on average mildly cognitively impaired (MMSE M = 22.3) and had a mild to moderate functional impairment (Barthel Index M = 50.4; HABAM M = 19.1). The Edmonton Frail Scale showed a mean of 7.4 and half of the patients (52.3%) had been assigned a care level. About 46.9% had a geriatric diagnosis, 3.0% had a diagnosis of dementia. According to DSM-V 19.2% of the patients had at least one main symptom of depression. The mean number of regularly taken drugs per patient was 8.2. Utilization of health care services prior to the hospital stay was rather low. On average, the sample showed 4.38 care related needs in general, of which 0.60 needs were unaddressed at the time of assessment.

**Conclusions:**

Descriptive analyses highlight an in-depth insight into impairments and different care needs of people with cognitive impairments. The results emphasize the need for gender-specific analyses as well as an increased attention to the heterogeneity of needs of people with cognitive impairments related to specific wards, settings and regions where they are admitted. Our results indicate also that people with cognitive impairments represent a high proportion of older patients in acute hospital care.

**Trial registration:**

The intersec-CM trial is registered at ClinicalTrials.gov (NCT03359408).

## Background

The hospital setting is a challenge for healthcare for older people [1]. Current figures indicate that older people visit the emergency unit in hospitals more often than younger people. In 2017, the age-specific number of hospital cases per 100,000 inhabitants in Germany was 49,945 for the age group of 65 years and older, while it was 23,470 cases per 100,000 inhabitants on average [2]. In addition to their somatic illnesses, many of these patients also face mental health problems. In general approximately 50% of older hospital patients have cognitive impairments, 27% suffer from delirium, 8–32% suffer from depressive symptoms, 21% suffer from apathy, and 9% from agitation and/or aggression [3].

According to a current epidemiological study in Germany, about 40% of patients aged 65 and older in hospitals show at least mild cognitive impairments [4]. Different degrees of cognitive impairment, impaired activities of daily living, receiving long-term care and unplanned hospital admission have been identified as significant patient-related risk factors for care challenges in the hospital setting [5]. The same risk factors are associated with the prescription of neuroleptics and specialist consultations and results in need for support from relatives [5]. In addition behavioral and psychological symptoms of dementia are common in the hospital setting and are associated with considerable distress in nursing staff, as well as a wide range of special treatments needs and additional behavioral and medical complications [6]. There is emerging evidence that interventions such as staff education, people with cognitive impairments related training and expertise, standardized care protocols and environmental modification can help to meet the needs of people with dementia in acute hospital settings [7]. Nurses can assist older people with dementia by encouraging evidence-based care practices [8]. Management strategies are called for to improve the situation for both patients and hospital staff [6]. The recent General Hospital Study (GHoSt) provides an detailed evidence-based overview of the situation in general hospitals in Germany [9].

One major challenge until today is, that cognitive impairments in the hospital are severely underdiagnosed. According to the German Federal Health Reporting Database (www.gbe-bund.de), there was a total of 19,952,735 patients treated in hospitals in Germany in 2017 [10]. According to this statistic *n* = 25,069 of those (0.13%) were diagnosed with neurodegenerative diseases including Alzheimer dementia (ICD-codes G30, G31) and *n* = 99,114 (0.50%) were diagnosed with an organic, including symptomatic, mental disorder (F00-F09, as this is not further detailed), this category includes different other types of dementia. These figures are based on secondary data that is documented for reimbursement purposes and thus addresses only a fraction of patients with cognitive impairments in routine care. Standardized and systematic assessment of people with cognitive impairments is restricted to very few dementia sensitive hospitals in Germany. For all others it remains a major challenge to meet the needs of people with cognitive impairment on a systematic level. There is an urgent need to improve routines for identification and provision of special care services for older patients with cognitive impairment and risk of delirium in general hospitals [11].

It is well established that admission to a hospital can worsen pre-existing cognitive problems, and can increase the risk for readmission, institutionalization or mortality [12–14]. These risks are aggravated by factors such as high age, comorbidities, malnutrition, low level of everyday functioning, depression and other mental disorders. All of these can be targeted during a hospital stay. Concepts for dementia-sensitive hospitals have been developed, systematic implementation in the German health care system, however, is challenging and positive examples are rare [15].

A recent scoping review indicates significant gaps in hospital care for older people with cognitive impairments and highlights the particular challenges at the interface of hospital care and ambulatory care [16]. For example, the hospital discharge is a process that needs to be prepared throughout the hospital stay and also should include care after discharge. Often, there is no contact person known for the time immediately after discharge so that care needs have to be organized by the people with cognitive impairment themselves or their informal caregivers and care gaps emerge [17, 18]. Care gaps lead to early institutionalization [19], increase the risk of unplanned re-admission to the hospital [20] and mortality [21]. Hospital discharge for people with cognitive impairments should be prepared for the transition to be as smooth as possible [18, 22]. In 2017, discharge management was adopted into German Social Law [23] and an expert standard has been defined [24]. However, while knowledge about care needs and associated factors in the ambulatory setting is improving [25], there is still sparse data on the needs of people with cognitive impairments in hospitals – and this lack limits any evidence based management that would support a smooth and seamless transition after discharge.

Thus, the aim of the present research is to describe and analyze their complex treatment needs of people with cognitive impairments in acute hospital care in Germany.

## Methods

### Study design

This analysis is part of the ongoing intersec-CM trial (Supporting elderly people with cognitive impairment during and after hospital stays with Intersectoral Care Management). Intersec-CM is a multisite, longitudinal, randomized controlled intervention trial (RCT) with two arms (intervention vs. “care as usual”) and four time points of data assessment (screening, baseline, follow-up 1, follow-up 2). Assignment to each arm was randomized after baseline assessment using computerized permuted blocks. This ensured that participants were distributed evenly between the two arms. Allocation to either the intervention or control group was conducted at the study center after the baseline assessment. Therefore the study staff was blind to the allocation during the initial assessment in the hospital and could not influence the allocation at all. Once the intervention had started full blinding of the study staff was not possible due to the type of intervention.

The aim of the ongoing study is to compare the effectiveness of an intervention in a group receiving the intervention (intersectoral care management) with a control group receiving “care as usual”. Three institutions participate in this trial, the Evangelisches Klinikum Bethel in Bielefeld/ North Rhine-Westphalia and the two sites of the University Medicine in Greifswald/ Mecklenburg-Western-Pomerania in Wolgast and Greifswald. At the Bielefeld site the recruiting medical specialties included neurology, trauma surgery, internal medicine (nephrology and gastroenterology). In Greifswald, the medical specialties comprised internal medicine and trauma surgery, and in Wolgast internal medicine and geriatrics. Ethical approval for this trial has been obtained from the Ethical Committee of the University Medicine Greifswald (Registry number: BB 159/17) and the Ethical Committee of the Chamber of Physicians Westphalia-Lippe (Registry number: 2017–688-b-S). The trial is registered at ClinicalTrials.gov (NCT03359408). The design of the study has been published in more detail elsewhere [26]. The present analysis is based on cross-sectional data of all participants at baseline.

Participants were enrolled in this study between 1st of November 2018 and end of March 2020. The recruitment process was standardized across sites. Specially trained study staff identified possible participants for the study after scanning their medical records for eligibility to the following inclusion criteria: age 70+, having lived at home prior to the index admission and living in the catchment area of the hospital. The list of eligible study participants was briefly discussed with routine care staff. Participants were excluded when routine staff indicated that (a) discharge was anticipated to be different than home (b) systematic screening procedure would be too burdensome for the patient and (c) anticipated stay would be too short. Then a standardized screening instrument (Mini Mental State Examination, MMSE 10–26) was used to detect possible cognitive impairment [27]. This approach was chosen based on a pretest and guided by the need to establish a procedure that could be implemented in routine care. A systematic screening of all patients had been decided to be inefficient and was perceived as interfering with routine care too much, which would have decreased motivation to cooperate. The limitations due to this recruitment procedure are discussed in the limitation section. To ensure the reliability of self-reported data we excluded patients with severe cognitive impairment. Patients provided written informed consent. We did not include any participants that were not consenting to participate (independent from their capacity to consent). Exclusion criteria were acute stroke as the primary reason for admission, any terminal disease, and nonsufficient German language skills. Since the treatment and care of older people with cognitive impairment is often dependent on informal caregivers, participants were asked to name their informal caregivers (e.g., spouse, child, friend), but providing an informal caregiver was not a necessary requirement for participation of the person with cognitive impairment. If an informal caregiver was available, they were invited as an independent study participant upon provision of written informed consent. After written informed consent was obtained, study staff began computer-based data collection at the time of baseline assessment by using standardized interviews. Collection of baseline data occurred on average 1.9 days after hospital admission (arithmetic mean, SD = 1.31).

### Sample

**Fig. 1:**
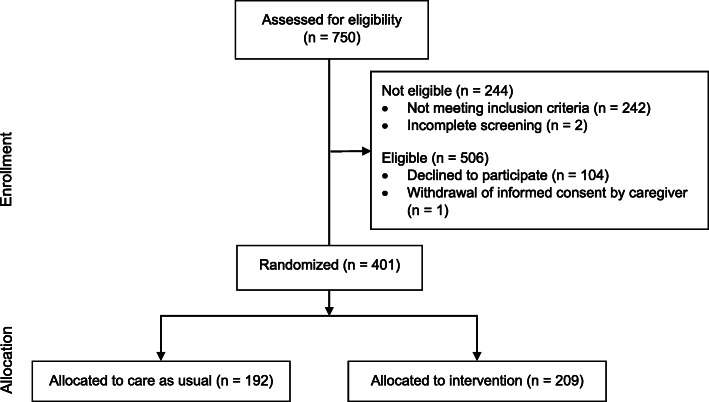
CONSORT diagram of enrollment and allocation in this study

Older people with cognitive impairments admitted to a general hospital with somatic illnesses were eligible for the study. Based on data from patient records people were identified as candidates for participation. A total of 750 patients were screened for cognitive impairment. Of these, 506 patients (67.5%) were eligible and of these *n* = 402 patients (79.4%) gave written informed consent to participate in the study. One patient dropped out of the study due to withdrawal of informed consent by a caregiver. We found no statistically significant differences between participants and eligible non-participants regarding the variables age (*t*(504) = − 1.444, *p* = .149), sex (χ2(1) = .001, *p* = .978) or cognitive status (*t*(502) = −.094, *p* = .925). The generation of the study population is described according to the Consolidated Standards of Reporting Trials (CONSORT) in Fig. 1.

### Procedures and instruments

For the present analysis, we assessed sociodemographic variables, clinical variables (cognitive status, functional status, status of balance and mobility, frailty, formal diagnosis of dementia, formal geriatrics diagnosis, level of impairment, depressive symptoms, delirium) and health care variables (utilization of health services, health care and intervention needs).

- Sociodemographic variables included age, sex, family status (single, married, divorced, widowed), living situation (alone/ not alone), and having children (yes/ no).
- Cognitive status was assessed using the German version of the Mini Mental State Exam (MMSE) [27, 28]. The MMSE provides a categorization differentiating “no indication” (score 27–30) “mild” (score 20–26), “moderate” (score 10–19) and “severe cognitive impairment” (score 0–9) as well as a total score which consists of items covering orientation, memory, attention, arithmetic, and language [29]..
- The functional status was assessed using the Barthel Index of Activities of Daily Living [30]. We utilized eight of the ten variables addressed in the Barthel Index, which were the items of help needed with feeding, grooming, bathing, dressing, toilet use and climbing stairs and the presence of stool and urinary incontinence. This yields a mean score of 0 to 70, where 0 indicates the highest possible impairment and 70 indicates the lowest possible impairment.
- The status of balance and mobility was assessed using the Hierarchical Assessment of Balance And Mobility (HABAM) [31] with the three domains balance, transfer and mobility. The assessment assigns a score to each domain and a total score, which indicates the highest level of performance in the three domains. For balance the score has a range from 0 to 21, thereby the value 0 marks no performance and 21 the highest level in balance. For transfer the range spans from 0 to 18 and for mobility from 0 to 26.
- Frailty was assessed using the Edmonton Frail Scale (EFS) [32]. This is a multidimensional measurement with nine dimensions. Each dimension is measured and scored separately and the sum score of all quantifies frailty. The frailty score ranges between 0 and 17 meaning the higher the score the frailer the participant. Due to the special situation of recruiting in a hospital setting, changes were needed with some dimensions. Furthermore to lessen the burden with a time consuming interview some items were replaced with items that were already part of the questionnaire, while staying as close to the EFS as possible. Cognition for example was measured using the MMSE instead of the clock-drawing test. Functional performance was replaced with the HABAM.
- For all patients who had provided the respective informed consent all formal medical diagnoses were retrieved from medical records. The presence of formal geriatric diagnosis, for example dizziness and giddiness or repeated falls, and formal diagnosis of dementia were analyzed for this paper.
- Level of impairment was defined according to the “care level (Pflegegrad)” used by the German care insurance for long-term care [33]. Each person is assigned to either none or one of five specific grades of care need. If a care level is assigned, each patient is categorized into one of five levels ranging from 1 to 5, with people in five needing the highest and in one the lowest level of care.
- Depressive symptoms were screened with the Patient Health Questionnaire (PHQ-2) [34]. The patients were asked how often they had been bothered by a) loss of interest or pleasure in activities and b) sadness during the last 2 weeks. These two variables represent the main indicators for a positive screening of depression according to DSM-V. The options to answer were 0 = never, 1 = single days, 2 = more than half of the days and 3 = nearly daily. If one of the items had a value of 3, the patient had a positive screening of depression.
- A possible delirium was assessed using the 4AT [35], which is a 4 question tool feasible for all patients, including those unable to speak, so that no patient is ‘unable to assess’. It consists of an item assessing level of alertness, a test of orientation, a test of attention, and an item discriminating acute change from a fluctuating course [36].
- For all patients pharmacological treatment information was retrieved from the medical records in the hospital. We summarized the total number of regularly taken drugs, prescription only, non-prescription and over-the-counter.
- Utilization of health care services was assessed by whether the person had used one or more of a list of services during the preceding 12 months (yes/no). Services asked for were hospital stays, rehabilitation, ambulatory care, all day and night care, short-term care, care counselling and additional care services.
- Health care needs were assessed using the Camberwell Assessment for Needs in the elderly (CANE) as validated questionnaire with sufficient psychometric properties [37, 38]. The CANE has been used extensively in various countries and has been validated for a German population of older people [39, 40]. The CANE assessment covers 25 dimensions, such as self-care or daytime activities, which can be adapted to fit the requirements of the study. 5 dimensions were not assessed: safety (deliberate self-harm, accidental self-harm, abuse/neglect), psychotic symptoms and physical health.

### Statistical analysis

In the present study, we provide descriptive statistics to analyze the study population at baseline. Metric variables are presented by means and standard deviations, nominal variables by categories and proportions. For the comparison between intervention and control group, we used Welch’s t-test, Chi-square test and Fisher’s exact test. For comparison between the hospitals one-way ANOVA was used. The statistical significance level was set to α < 0.05. Not all variables in the baseline assessment could be assessed in all participants. The descriptive statistics provide the respective number of participants for each assessment tool. We used IBM SPSS for the statistical analyses.

## Results

The final sample of *n* = 401 patients is on average 82.4 years old (arithmetic mean, SD = 6.1) and 63.3% female. About 61.9% of the patients reported to live alone, 5.8% had no caregiver available at the time of the baseline assessment. The family status of 36.3% of the patients was married, 53.5% of them were widowed. A few were single (4.3%) or divorced (5.9%). The majority of the participants indicated that they have children (90.0%). There were no statistically significant differences between the intervention and control group in respect to sociodemographic variables. For detailed information, see Table 1.

**Table 1:** Sociodemographic variables

	Total sample (*n* = 401)		Control group (*n* = 192)		Intervention group (*n* = 209)		*p*-value*
Age, mean (years) (SD)	82.4	(6.1)	82.4	(6.1)	82.5	(6.0)	.764
Sex (female), n (%)	254	63.3%	121	63.0%	133	63.6%	.490
Family status	*n* = 391		*n* = 185		*n* = 206		
Single	17	4.3%	8	4.3%	9	4.4%	.460
Married	142	36.3%	70	37.8%	72	35.0%	
Divorced	23	5.9%	14	7.6%	9	4.4%	
Widowed	209	53.5%	93	50.3%	116	56.3%	
Living alone	*n* = 394		*n* = 188		*n* = 206		
(yes), n (%)	244	61.9%	113	60.1%	131	63.6%	.272
Having children	*n* = 391		*n* = 185		*n* = 206		
(yes), n (%)	350	90.0%	170	92.9%	180	87.4%	.050

* Statistically significant difference between control and intervention group on a level of significance of α < 0.05; different n’s due to missing data

The distribution of the patients over the hospitals and medical specialty is shown in Tables 2 and 3.

**Table 2:** Distribution over the medical specialties

Medical specialties, n (%)	Total sample (*n* = 401)		Control group (*n* = 192)		Intervention group (*n* = 209)	
Internal medicine	59	14.7%	29	15.1%	30	14.4%
Neurology	30	7.5%	12	6.3%	18	8.6%
Geriatrics	106	26.4%	50	26.0%	56	26.8%
Trauma surgery	40	10.0%	17	8.9%	23	11.0%
Nephrology	81	20.2%	46	24.0%	35	16.7%
Gastroenterology	85	21.2%	38	19.8%	47	22.5%

**Table 3:** Distribution over the hospitals

Hospital, n (%)	Total sample (*n* = 401)		Control group (*n* = 192)		Intervention group (*n* = 209)	
1	200	49.9%	100	52.1%	100	47.8%
2	62	15.5%	24	12.5%	38	18.2%
3	139	34.7%	68	35.4%	71	34.0%

Patients in our final sample reached an average MMSE score of 22.3 (arithmetic mean), indicating a generally milder cognitive impairment in our study population. A total of 80.5% of the population were categorized with “mild cognitive impairment” according to the MMSE, 19.5% of the patients had “moderate cognitive impairment”. The functional status (Barthel Index) ranged from 5 to 70, with 70 indicating no functional impairment. Our study population shows a mean score of 50.4, which indicates a mild to moderate functional impairment. The hierarchical assessment of balance and mobility (HABAM) yields an average score of 19.1. The score indicates the highest value on one of the three dimensions balance, transfer and mobility. To look at each dimension separately, we computed mean scores for balance, transfer and mobility. The mean score for balance was 12.7, for transfer 14.5 and for mobility 16.0. These means show a moderate impairment for all three dimensions.

About 46.9% of the study population had a geriatric diagnosis, 12 patients (3.0%) had a diagnosis of dementia. A possible delirium was determined in 8.0% of the sample. The Edmonton Frail Scale shows a mean frailty score of 7.4 indicating a vulnerable sample. In *n* = 77 (19.2%) patients of the sample, one of the main symptoms of depression according to DSM-V was found. The mean number of regularly taken drugs as part of the pharmacological treatment is 8.2 in the sample. For none of the described variables a statistically significant difference between intervention and control group was found (see Table 4).

**Table 4:** Clinical variables in comparison between study groups

	Total sample (*n* = 401)		Control group (*n* = 192)		Intervention group (*n* = 209)		*p*-value
*Cognitive status (MMSE)*	*n* = 401		*n* = 192		*n* = 209		
Score, (10–26), mean (SD)	22.2	(3.6)	22.0	(3.6)	22.3	(3.7)	.374
Mild cognitive impairment (score 20–26), n (%)	323	80.5%	154	80.2%	169	80.9%	.484
Moderate cognitive impairment (score 10–19), n (%)	78	19.5%	38	19.8%	40	19.1%	
*Functional Status (Barthel)*	*n* = 400		*n* = 191		*n* = 209		
Score, (5–70), mean (SD)	50.4	(15.4)	49.9	(15.6)	50.8	(15.2)	.541
*HABAM*	*n* = 401		*n* = 192		*n* = 209		
Score (0–26), mean (SD)	19.1	(5.9)	19.0	(5.7)	19.3	(6.1)	.556
Balance^a^ (0–21), mean (SD)	12.7	(7.0)	12.7	(6.9)	12.7	(7.1)	.994
Transfer^b^ (0–18), mean (SD)	14.5	(5.0)	14.5	(4.9)	14.5	(5.1)	.959
Mobility^c^ (0–26),mean (SD)	16.0	(7.8)	16.0	(7.5)	16.0	(8.1)	.978
*Edmonton Frailty Index*	*n* = 397		*n* = 188		*n* = 209		
Score, (2–15), mean (SD)	7.4	(2.5)	7.4	(2.5)	7.3	(2.6)	.675
*Level of impairment*	*n* = 396		*n* = 188		*n* = 208		
Yes, n (%)	197	50.3%	106	56.4%	91	43.8%	.008*
Care level							
None, n (%)	199	51.0%	82	44.8%	117	56.5%	.084
1, n (%)	29	7.4%	15	8.2%	14	6.8%	
2, n (%)	88	22.6%	41	22.4%	47	22.7%	
3, n (%)	63	16.2%	38	20.8%	25	12.1%	
4, n (%)	10	2.6%	6	3.3%	4	1.9%	
5, n (%)	1	0.3%	1	0.5%	0	0.0%	
*Diagnosis of dementia (ICD-10)*	*n* = 401		*n* = 192		*n* = 209		
Yes, n (%)	12	3.0%	8	4.2%	4	1.9%	.152
*Geriatric Diagnosis (ICD-10)*	*n* = 401		*n* = 192		*n* = 209		
Yes, n (%)	188	46.9%	89	46.4%	99	47.4%	.459
*Delirium possible*	*n* = 387		*n* = 185		*n* = 202		
Yes, n (%)	31	8.0%	16	8.6%	15	7.4%	.399
*Depression*	*n* = 401		*n* = 192		*n* = 209		
At least one main symptom of depression according to DSM-V, n (%)	77	19.2%	38	19.8%	39	18.7%	.436
*Pharmacological treatment*	*n* = 398		*n* = 191		*n* = 207		
Total number of regularly taken drugs, mean (SD)	8.2	(3.8)	8.6	(4.0)	7.9	(3.7)	.078
*Utilization of health care services (preceding year)*							
Hospital stay^d^							
One or two hospital stays^d^, n (%)	293	74.9%	144	77.0%	149	73.0%	.216
More than two hospital stays^d^, n (%)	98	25.1%	43	23.0%	55	27.0%	
Rehabilitation^e^, n (%)	46	11.8%	23	12.6%	23	11.1%	.372
Ambulatory care^f^, n (%)	152	38.8%	85	45.9%	67	32.4%	.004*
Total formal care (all day & night care)^g^, n (%)	13	3.3%	10	5.5%	3	1.5%	.027*
Short term care^h^, n (%)	28	7.3%	20	10.9%	8	4.0%	.007*
Care counselling^i^, n (%)	75	21.2%	45	27.4%	30	15.8%	.005*
Additional care services^j^, n (%)	79	21.6%	46	26.0%	33	17.5%	.032*
*CANE*	*n* = 396		*n* = 188		*n* = 208		
Sum needs overall (0–15), mean (SD)	4.38	(0.14)	4.65	(0.22)	4.13	(0.19)	.074
Sum unaddressed needs (0–8), mean (SD)	0.60	(0.05)	0.60	(0.08)	0.59	(0.07)	.927

* Statistically significant difference between control and intervention group on a level of significance of α < 0.05

^a^Total *n* of sample = 395; *n* of Control Group = 190; *n* of Intervention Group = 205

^b^Total *n* of sample = 399; *n* of Control Group = 192; *n* of Intervention Group = 207

^c^Total *n* of sample = 399; *n* of Control Group = 190; *n* of Intervention Group = 209

^d^Total *n* of sample = 391; *n* of Control Group = 187; *n* of Intervention Group = 204

^e^Total *n* of sample = 390; *n* of Control Group = 182; *n* of Intervention Group = 208

^f^Total *n* of sample = 392; *n* of Control Group = 185; *n* of Intervention Group = 207

^g^Total *n* of sample = 389; *n* of Control Group = 183; *n* of Intervention Group = 206

^h^Total *n* of sample = 386; *n* of Control Group = 184; *n* of Intervention Group = 202

^i^Total *n* of sample = 354; *n* of Control Group = 164; *n* of Intervention Group = 190

^j^Total *n* of sample = 366; *n* of Control Group = 177; *n* of Intervention Group = 189; different n’s due to missing data

A total of *n* = 197 patients had been assigned a care level (52.3%). The most frequent care level was level 2 (46.1%), followed by level 3 (33.0%). There was a statistically significant difference between the control and intervention group. In the control group more patients had been assigned a care level (56.4% versus 43.8%). The differences in the single care levels between the study groups are shown in Table 4.

Analyzing utilization of health care professionals in the last 12 months, 74.9% had one or two hospital stays, 25.1% visited the hospital more often. Rehabilitation was used by 11.8% of the sample. We found no statistical differences for these variables between the groups. The frequency of using ambulatory care was 38.8% in the total sample. In the control group, 45.9% of the patients used ambulatory care, in the intervention group just 32.4%, resulting in a statistically significant difference. Considering total formal care, 3.3% of the population had all day and night care. Patients in the control group used total formal care more frequently than the intervention group (5.5% versus 1.5%), a statistically significant difference. 10.9% of the control group utilized short-term care, contrasting with 4.0% of the intervention group (7.3% in total), resulting in a statistically significant difference. The frequency of using care counselling was 21.2% in the whole sample. In the control group, 27.4% of the patients received previous care counselling, but just 15.8% of the patients in the intervention group, a statistically significant difference. There was also a statistically significant difference between the intervention and control group in respect to the utilization of additional care services. A larger proportion of patients in the control group used additional care services than the intervention group (26.0% versus 17.5%). There was no statistically significant difference between the study groups for the sum of needs, these ranged from 0 to 15 needs overall and 0 to 8 unaddressed needs. On average, the sample shows 4.38 needs in general, of which 0.60 needs are unaddressed.

The comparison between the hospital recruiting sites shows that 63.5% of the patients in hospital 3 and 65.8% of the patients in hospital 1 live alone, but just 45.9% of the patients in hospital 2, resulting in a statistically significant difference. The sites also differed in respect to several patients’ clinical variables. Furthermore differences between the hospitals were found in the number of the needs overall as well as the unmet needs. The participants recruited in hospital 1 showed significantly less unmet needs. There was, however, no difference in any other sociodemographic variable. For details, see Table 5.

**Table 5:** Clinical variables in comparison between the hospitals

	Hospital 1 (*n* = 200)		Hospital 2 (*n* = 62)		Hospital 3 (*n* = 139)		*p*-value
*Cognitive status (MMSE)*	*n* = 200		*n* = 62		*n* = 139		
Score, (10–26), mean (SD)	22.9	(3.1)	21.6	(3.9)	21.4	(4.1)	<.001*
Mild cognitive impairment (score 20–26), n (%)	169	84.5%	50	80.6%	104	74.8%	.086
Moderate cognitive impairment (score 10–19), n (%)	31	15.5%	12	19.4%	35	25.2%	
*Functional Status (Barthel)*	*n* = 200		*n* = 62		*n* = 138		
Score, mean (SD)	52.6	(15.3)	55.7	(11.8)	44.9	(15.4)	<.001*
*HABAM*	*n* = 200		*n* = 62		*n* = 139		
Score (0–26), mean (SD)	20.0	(5.7)	21.8	(4.8)	16.7	(5.8)	<.001*
Balance^a^ (0–21), mean (SD)	16.4	(5.7)	8.1	(7.1)	9.4	(5.8)	<.001*
Transfer^b^ (0–18), mean (SD)	15.9	(4.3)	14.2	(5.4)	12.7	(5.3)	<.001*
Mobility^c^ (0–26), mean (SD)	18.9	(6.3)	14.2	(9.3)	12.6	(7.4)	<.001*
*Edmonton Frailty Index*	*n* = 198		*n* = 60		*n* = 121		
Score, (2–15), mean (SD)	6.6	(2.3)	6.2	(1.8)	9.0	(2.2)	<.001*
*Level of impairment*	*n* = 196		*n* = 62		*n* = 138		
Yes, n (%)	100	51.0%	23	37.1%	74	53.6%	.085
Care Level							
None, n (%)	96	50.3%	39	63.9%	64	46.4%	.020*
1, n (%)	12	6.3%	8	13.1%	9	6.5%	
2, n (%)	43	22.5%	9	14.8%	36	26.1%	
3, n (%)	32	16.8%	4	6.6%	27	19.6%	
4, n (%)	8	4.2%	0	0.0%	2	1.4%	
5, n (%)	0	0.0%	1	1.6%	0	0.0%	
*Diagnosis of dementia (ICD-10)*	*n* = 200		*n* = 62		*n* = 139		
Yes, n (%)	4	2.0%	1	1.6%	7	5.0%	.214
*Geriatric Diagnosis (ICD-10)*	*n* = 200		*n* = 62		*n* = 139		
Yes, n (%)	76	38.0%	8	12.9%	104	74.8%	<.001*
*Delirium possible*	*n* = 200		*n* = 50		*n* = 137		
Yes, n (%)	9	4.5%	2	4.0%	20	14.6%	.002*
*Depression*	*n* = 200		*n* = 62		*n* = 139		
At least one main symptom of depression according to DSM-V, n (%)	17	8.5%	11	17.7%	49	35.3%	<.001*
*Pharmacological treatment*	*n* = 197		*n* = 62		*n* = 139		
Total number of regularly taken drugs, mean (SD)	7.5	(3.8)	7.2	(4.4)	9.8	(3.2)	<.001*
*Utilization of health care services (preceding year)*							
Hospital stay^d^							
One or two hospital stays^d^, n (%)	158	79.8%	48	80.0%	87	65.4%	.008*
More than two hospital stays^d^, n (%)	40	20.2%	12	20.0%	46	34.6%	
Rehabilitation^e^, n (%)	28	14.4%	1	1.6%	17	12.6%	.024*
Ambulatory care^f^, n (%)	67	34.2%	18	29.5%	67	49.6%	.005*
Total formal care (all day & night care) ^g^, n (%)	5	2.6%	1	1.6%	7	5.3%	.287
Short term care^h^, n (%)	22	11.3%	0	0.0%	6	4.6%	.004*
Care counselling^i^, n (%)	53	28.8%	9	15.8%	13	11.5%	.001*
Additional care services^j^, n (%)	23	12.2%	1	1.7%	55	47.0%	<.001*
*CANE*	*n* = 196		*n* = 62		*n* = 138		
Sum needs overall (0–15), mean (SD)	3.60	(0.17)	5.19	(0.47)	5.13	(0.25)	<.001*
Sum unaddressed needs (0–8), mean (SD)	0.21	(0.05)	1.29	(0.20)	0.83	(0.08)	<.001*

* Statistically significant difference between the hospitals on a level of significance of α < 0.05

^a^Total *n* of sample = 395; *n* of hospital 1 = 200; *n* of hospital 2 = 62; *n* of hospital 3 = 133

^b^Total *n* of sample = 399; *n* of hospital 1 = 200; *n* of hospital 2 = 61; *n* of hospital 3 = 138

^c^Total *n* of sample = 399; *n* of hospital 1 = 200; *n* of hospital 2 = 62; *n* of hospital 3 = 137

^d^Total *n* of sample = 391; *n* of hospital 1 = 198; *n* of hospital 2 = 60; *n* of hospital 3 = 133

^e^Total *n* of sample = 390; *n* of hospital 1 = 194; *n* of hospital 2 = 61; *n* of hospital 3 = 135

^f^Total *n* of sample = 392; *n* of hospital 1 = 196; *n* of hospital 2 = 61; *n* of hospital 3 = 135

^g^Total *n* of sample = 389; *n* of hospital 1 = 196; *n* of hospital 2 = 61; *n* of hospital 3 = 132

^h^Total *n* of sample = 386; *n* of hospital 1 = 194; *n* of hospital 2 = 61; *n* of hospital 3 = 131

^i^Total *n* of sample = 354; *n* of hospital 1 = 184; *n* of hospital 2 = 57; *n* of hospital 3 = 113

^j^Total *n* of sample = 366; *n* of hospital 1 = 189; *n* of hospital 2 = 60; *n* of hospital 3 = 117; different n’s due to missing data

There is a statistically significant sex difference in age and living status in the sample; female patients were older than males (83.0 versus 81.5 years) and lived alone more often (63.6% versus 34.7%). A difference in family status was not found. These differences are shown in Table 6.

**Table 6:** Sociodemographic variables in comparison between sexes

	Total sample (*n* = 401)		Male (*n* = 147)		Female (*n* = 254)		*p*-value
Age, mean [years] (SD)	82.4	(6.1)	81.5	(5.9)	83.0	(6.1)	.013*
Family status	*n* = 391		*n* = 142		*n* = 249		
Single	17	4.3%	8	5.6%	9	3.6%	<.001*
Married	142	36.3%	88	62.0%	54	21.7%	
Divorced	23	5.9%	5	3.5%	18	7.2%	
Widowed	209	53.5%	41	28.9%	168	67.5%	
Living alone	*n* = 391		*n* = 142		*n* = 249		
(yes), n (%)	244	61.9%	50	34.7%	131	63.6%	<.001*
Having children	*n* = 391		*n* = 140		*n* = 251		
(yes), n (%)	351	89.8%	129	92.1%	222	88.4%	.163

* Statistically significant difference between control and intervention group on a level of significance of α < 0.05; different n’s due to missing data

Furthermore, there is a sex difference in clinical variables (see Table 7). Regarding geriatric diagnoses and a possible delirium, more males had these diagnoses. For utilization of health care services, female patients used ambulatory care (42.6% versus 31.9%) and short-term care (9.3% versus 3.6%) more often. No other variable of utilization showed a statistically significant sex difference. Regarding the assessment of needs, male participants indicated more unmet needs, than the female participants (0.76 vs. 0.50) while showing no statistically significant difference in the sum of needs overall.

**Table 7:** Clinical variables in comparison between sexes

	Total sample (*n* = 401)		Male (*n* = 147)		Female (*n* = 254)		*p*-value
*Cognitive status (MMSE)*	*n* = 401		*n* = 147		*n* = 254		
Score, (10–26), mean (SD)	22.2	(3.6)	21.9	(4.0)	22.3	(3.5)	.273
Mild cognitive impairment (score 20–26), n (%)	323	80.5%	116	78.9%	207	81.5%	.307
Moderate cognitive impairment (score 10–19), n (%)	78	19.5%	31	21.1%	47	18.5%	
*Functional Status (Barthel)*	*n* = 400		*n* = 146		*n* = 254		
Score, mean (SD)	50.4	(15.4)	49.1	(16.1)	51.1	(14.9)	.196
*HABAM*	*n* = 401		*n* = 147		*n* = 254		
Score (0–26), mean (SD)	19.1	(5.9)	19.3	(6.3)	19.1	(5.7)	.762
Balance^a^ (0–21), mean (SD)	12.7	(7.0)	12.3	(7.3)	13.0	(6.9)	.300
Transfer^b^ (0–18), mean (SD)	14.5	(5.0)	14.6	(5.0)	14.5	(5.1)	.812
Mobility^c^ (0–26),mean (SD)	16.0	(7.8)	15.9	(8.2)	16.1	(7.6)	.850
*Edmonton Frailty Index*	*n* = 397		*n* = 145		*n* = 252		
Score, (2–15), mean (SD)	7.4	(2.5)	7.6	(2.4)	7.2	(2.5)	.136
*Level of impairment*	n = 396		*n* = 144		*n* = 252		
Yes, n (%)	197	50.3%	64	44.4%	133	52.8%	.068
Care level							
None, n (%)	199	51.0%	80	56.3%	119	48.0%	.338
1, n (%)	29	7.4%	9	6.3%	20	8.1%	
2, n (%)	88	22.6%	24	16.9%	64	25.8%	
3, n (%)	63	16.2%	26	18.3%	37	14.9%	
4, n (%)	10	2.6%	3	2.1%	7	2.8%	
5, n (%)	1	0.3%	0	0.0%	1	0.4%	
*Diagnosis of dementia (ICD-10)*	*n* = 401		*n* = 147		*n* = 254		
Yes, n (%)	12	3.0%	2	1.4%	10	3.9%	.122
*Geriatric Diagnosis (ICD-10)*	*n* = 401		*n* = 147		*n* = 254		
Yes, n (%)	188	46.9%	76	51.7%	112	44.1%	.086
*Delirium possible*	*n* = 387		*n* = 140		*n* = 247		
Yes, n (%)	31	8.0%	19	13.6%	12	4.9%	.003*
*Depression*	*n* = 401		*n* = 147		*n* = 254		
At least one main symptom of depression according to DSM-V, n (%)	77	19.2%	27	18.4%	50	19.7%	.427
*Pharmacological treatment*	*n* = 398		*n* = 145		*n* = 253		
Total number of regularly taken drugs, mean (SD)	8.2	(3.8)	8.7	(3.6)	8.0	(4.0)	.064
*Utilization of health care services (preceding year)*							
Hospital stay^d^							
One or two hospital stays^d^, n (%)	293	74.9%	103	72.0%	190	76.6%	.187
More than two hospital stays^d^, n (%)	98	25.1%	40	28.0%	58	23.4%	
Rehabilitation^e^, n (%)	46	11.8%	13	9.2%	33	13.3%	.153
Ambulatory care^f^, n (%)	152	38.8%	45	31.9%	107	42.6%	.023*
Total formal care (all day & night care)^g^, n (%)	13	3.3%	2	1.4%	11	4.4%	.093
Short term care^h^, n (%)	28	7.3%	5	3.6%	23	9.3%	.025*
Care counselling^i^, n (%)	75	21.2%	28	20.9%	47	21.4%	.514
Additional care services^j^, n (%)	79	21.6%	27	20.1%	52	22.4%	.356
*CANE*	*n* = 396		*n* = 147		*n* = 254		
Sum needs overall (0–15), mean (SD)	4.38	(0.14)	4.17	(0.25)	4.50	(0.17)	.269
Sum unaddressed needs (0–8), mean (SD)	0.60	(0.05)	0.76	(0.11)	0.50	(0.05)	.033*

* Statistically significant difference between sex on a level of significance of α < 0.05

^a^Total *n* of sample = 395; *n* of male = 144; *n* of female = 251

^b^Total *n* of sample = 399; *n* of male = 146; *n* of female = 253

^c^Total *n* of sample = 399; *n* of male = 146; *n* of female = 253

^d^Total *n* of sample = 391; *n* of male = 143; *n* of female = 248

^e^Total *n* of sample = 390; *n* of male = 141; *n* of female = 249

^f^Total *n* of sample = 392; *n* of male = 141; *n* of female = 251

^g^Total *n* of sample = 389; *n* of male = 141; *n* of female = 248

^h^Total *n* of sample = 386; *n* of male =140; *n* of female = 246

^i^Total *n* of sample = 354; *n* of male = 134; *n* of female = 220

^j^Total *n* of sample = 366; *n* of male = 134; *n* of female = 232; different n’s due to missing data

## Discussion

The aim of the present study was to describe and analyze the situation of people with cognitive impairments in acute care hospitals and to identify care needs that need support for returning home and therefore into the ambulatory health care setting. In accordance with other studies [4], our results indicate that people with cognitive impairments represent a high proportion of old people in acute hospital care, as about two thirds of the patients screened for eligibility were cognitively impaired. This percentage is much higher than assumed in the literature [4]. Our recruiting method was based on preselection using medical records and worked as an efficient case-finding procedure. This approach spares much of the effort for random screening which makes it more feasible in the daily clinical work. In our final sample under examination about 80% of our patients had been mildly cognitive impaired, the remaining 20% had a moderate impairment. A possible delirium was found in 8% of the patients. Surprisingly, only 3% of the patients had received a formal diagnosis of dementia which should have been more common in this group of patients. The absence of dementia diagnoses indicates that cognitive impairment is not a priority of clinical attention. Our results fit into previous research that shows similar distributions of cognitive impairment [41, 42] and show the benefit of systematic identification of people with cognitive impairments in the clinical setting. The intersec-CM experience confirms that people with cognitive impairments can be detected under routine care conditions and that the identification of cognitive impairments can support the provision of appropriate treatment and care.

To the best of our knowledge, this study is the first to deliver a most comprehensive description of people with cognitive impairments in three acute hospitals in Germany. It highlights the complexity of care needed to meet the individual patient’s needs which are determined by the presence of a multitude of impairments besides the initial reason for hospital admission. The majority of the patients shows mild to moderate functional impairment and limitations in mobility, balance and transfer, a syndrome that requires more extensive care. The level of impairment shows that almost half of our sample had a care level prior to admission. The majority of those have been assigned the second care level, which refers a considerably reduced independence. For the pharmacological treatment our sample shows an average of eight regularly taken drugs. This high number reinforces the significance of an adequate medication review.

Our sample is, due to physical decline, frail and vulnerable to a subsequent further deterioration of health and therefore prone to further loose independency. The screening for a possible depression shows that about 20% of our sample had at least one of the two main symptoms of a depression according to DSM-V. Almost 50% of the sample had geriatric diagnoses. The utilization of health care services shows that the majority of the sample had one or two hospital stays in the year preceding the index admission, however, only few had more than two stays. Ambulatory care and care counselling are the most frequently used health care services, which again illustrates the need for a professional care transition. The assessment of care needs yield roughly four needs, with less than one unaddressed need per person. This is lower than assumed based on previous studies [37]. However, this assessment only reflects the needs surveyed by the CANE instrument. For our intervention we assessed several other needs in addition to the CANE, such as the medication review or the need for a review of the adequacy of the care level. The true number of interventable needs is therefore greater [37, 43].

Our results also highlight the need to look deeper into sex-differences in aging and provision of care. Our final sample under examination comprised about two-thirds females. Women in our sample are, similar to previous research [44], more likely to be widowed while men are more likely to be married. This is also reflected in the patients’ life situation. Almost twice as many female patients live alone [45]. Furthermore, women are on average 1.5 years older than men. Males had more often a geriatric diagnosis and a possible delirium, which fits into current discussions about male sex as a risk factor for a delirium [46]. This goes along with our finding of higher numbers of unaddressed care needs in men, since especially a geriatric diagnosis is usually linked to more care needs. The difference between men and women with respect to the number of needs was found similar as in other studies [37]. On the other hand, women used some health care services more often than men, for example ambulatory care. Possibly this finding can be explained by the higher age of the women in the sample. Due to women having a higher life expectancy and typically being younger than their spouses, women outlive their spouses in the majority of cases. This explains the observed differences in marital status as well as in the living situation. Through after-effects this also explains to some extent the differences in utilization of health-care services; as long as there are both partners, the care dyad often functions effectively up to a very high care need of one or even both partners. Taking into consideration that often the wife is available when the male spouse needs care, use of professional care is less prevalent among older men. When the woman needs care later in her life, the male spouse is often either dependent himself or already deceased, so there is more need for professional care such as ambulatory care among women. These effects are enhanced by the known factor of the gender care gap. In summary, the found differences for gender are explainable and are in line with previous research.

We observed considerable variability between people with cognitive impairments in the utilization of health care services. This differs greatly between the different settings and locations, be that urban/rural, east/west or medical specialty. It can be assumed that this is at least partly due to a differing availability of health care services, as it is known that the health care network is usually less tight in rural areas. In addition to this discrepancy, there is still some imbalance between the west and the east of Germany, with East Germany being less well supplied. However, these factors do not explain the observed differences between the hospitals, the medical specialty is prone to play into them because only in one of the hospitals a specialized geriatric ward could be included in our study. The patients admitted to this ward are more likely in need for care and often are worse in their overall health. The causes for these intertwined effects need further examination.

The generalizability of our result is limited due to our recruitment procedure. There might be a selection bias. Rather than screening the entire population of hospital patients for the eligibility criteria, we also relied on advise and judgement of the routine care staff. Their judgement was not quantitatively assessed, systematically documented or validated. Therefore we might have missed patients with moderate cognitive impairment. However, the study needed to be conducted in a routine care setting with routine care conditions. As such there was a trade-off between being able to conduct a rigid, standardized screening procedure aiming at representativity and/ or ensuring a high degree of cooperation from routine care staff without implementing procedures to be perceived as additional work load or interfering with routine business. With this study aiming at supporting patients with cognitive impairment and needing the (unpaid) support of routine staff, we chose the latter approach. Another limitation relates to the identification of people with cognitive impairment. We could not use guideline-based diagnostics, so there is the possibility that the number of identified people with cognitive impairments is somewhat over- or underestimated. A last point to be mentioned here is the validity of the assessment. Some questions, e.g. about utilization of health care services, were answered retrospectively by people with cognitive impairments, so that the results must be considered with due caution.

## Conclusion

Our results show an urgent need to improve identification of people with cognitive impairments in acute care hospitals. With our descriptive analyses, we provide an in-depth description of impairments and a variety of care needs. We highlight the need for sex-specific analyses as well as the heterogeneity of care needs between people with cognitive impairments, related to wards, settings and regions where they are admitted to. Longitudinal analyses are necessary to analyze different health outcomes based on patient related and contextual differences at baseline. The goal is to identify risk factors and determinants for effective diagnose, treatment and rehabilitation of people with cognitive impairments in the acute hospital – and develop specific interventions to ameliorate the present challenges.

## Data Availability

The datasets generated and analyzed during the current study are not publicly available due to legal reasons but are available from the corresponding author on reasonable request.

## Abbrevations

CANE: Camberwell Assessment for Needs in the elderly
HABAM: Hierarchical Assessment of Balance And Mobility
MMSE: Mini Mental State Exam
RCT: Randomized controlled trial
